# The Validity and Responsiveness of Isometric Lower Body Multi-Joint Tests of Muscular Strength: a Systematic Review

**DOI:** 10.1186/s40798-017-0091-2

**Published:** 2017-06-19

**Authors:** David Drake, Rodney Kennedy, Eric Wallace

**Affiliations:** 1 School of Sport, Ulster University, Jordanstown, Shore Road, Newtownabbey, Co. Antrim BT37 0QB UK; 2 Ulster Rugby, Kingspan Stadium, 134 Mount Merrion Avenue, Belfast, Co. Antrim BT6 0FT UK; 3 Sport and Exercise Sciences Research Institute, Ulster University, Jordanstown, Shore Road, Newtownabbey, Co. Antrim BT37 0QB UK

## Abstract

**Background:**

Researchers and practitioners working in sports medicine and science require valid tests to determine the effectiveness of interventions and enhance understanding of mechanisms underpinning adaptation. Such decision making is influenced by the supportive evidence describing the validity of tests within current research. The objective of this study is to review the validity of lower body isometric multi-joint tests ability to assess muscular strength and determine the current level of supporting evidence.

**Methods:**

Preferred Reporting Items for Systematic Reviews and Meta-Analysis (PRISMA) guidelines were followed in a systematic fashion to search, assess and synthesize existing literature on this topic. Electronic databases such as Web of Science, CINAHL and PubMed were searched up to 18 March 2015. Potential inclusions were screened against eligibility criteria relating to types of test, measurement instrument, properties of validity assessed and population group and were required to be published in English. The Consensus-based Standards for the Selection of health Measurement Instruments (COSMIN) checklist was used to assess methodological quality and measurement property rating of included studies. Studies rated as fair or better in methodological quality were included in the best evidence synthesis.

**Results:**

Fifty-nine studies met the eligibility criteria for quality appraisal. The ten studies that rated fair or better in methodological quality were included in the best evidence synthesis. The most frequently investigated lower body isometric multi-joint tests for validity were the isometric mid-thigh pull and isometric squat. The validity of each of these tests was strong in terms of reliability and construct validity. The evidence for responsiveness of tests was found to be moderate for the isometric squat test and unknown for the isometric mid-thigh pull. No tests using the isometric leg press met the criteria for inclusion in the best evidence synthesis.

**Conclusions:**

Researchers and practitioners can use the isometric squat and isometric mid-thigh pull with confidence in terms of reliability and construct validity. Further work to investigate other validity components such as criterion validity, smallest detectable change and responsiveness to resistance exercise interventions may be beneficial to the current level of evidence.

**Electronic supplementary material:**

The online version of this article (doi:10.1186/s40798-017-0091-2) contains supplementary material, which is available to authorized users.

## Key Points

- Isometric mid-thigh pulls and squats are reliable tests that can differentiate between strength ability of participants. These tests can be utilized by both male and females independent of their training status or experience.
- The isometric squat test can be used to track progress in muscular strength over time.
- Test protocols should be carefully considered and documented when using isometric multi-joint tests to ensure repeatability, such as joint angle at which testing occurred.
- Commonly isometric multi-joint tests are 5 s in duration, with two to three trials allowed and 3–5 min recovery between tests.

## Background

Testing of specific strength capabilities is a critical aspect of understanding the strategies that best enhance muscular strength [1, 2]. In assessing strength, Tillin et al. [3] recommended multi-joint rather than single-joint testing due to the specific neural and mechanical conditions in athletic performance tasks such as sprinting and jumping. Additionally, Gentil et al. [4] found no increased motor unit recruitment in single- versus multi-joint muscle actions. Isoinertial tests such as repetition maximum back-squats are frequently used to test lower body multi-joint strength [5–7]. Whilst isoinertial tests are common, associated methodological considerations challenge their validity to assess changes in muscular strength [8]. These considerations include approaches to squat depth, technical skill required to complete the range of movement under high external load as well as the number of trials required to build up to a maximal test load [9–12]. Previous research has reported that isoinertial tests lack practicality due to limitations in using isoinertial repetition maximum tests with certain populations, such as novice, elderly or functionally limited participants [6, 8, 13].

It is suggested that isometric multi-joint tests (IMJT) provide a time efficient assessment of muscular strength [14, 15] that allows less interruption to training compared to isoinertial repetition maximum testing. Given the potential practical merit, it is important to understand the overall validity of IMJTs. The predominance of research to date investigating IMJTs has assessed their specific correspondence to dynamic performance tasks, such as jump height [3], change of direction [16] and sprint performance [17]. However, this work does not enhance understanding of IMJTs validity as a measure of muscular strength. Therefore, research attention is required to evaluate the evidence directly investigating validity properties of IMJTs as an assessment tool to evaluate muscular strength.

Validity of a test refers to the degree to which it measures what it intends. Many different properties of validity can be assessed to examine the efficacy of a test. These properties include face and content validity, based on a judgement that the test is likely to measure the intended construct and that the test adequately represents the construct of interest [18]. Criterion validity is defined as the extent a test adequately reflects scores on a ‘gold standard’ test measuring the same construct [19]. Construct validity is the level to which the test measures the intended construct and the inference that can therefore be made from the scores. Construct convergent/discriminant validity relate to the extent to which test scores correlate/or not with scores on another test of the same construct. Construct validity for known groups is the degree test scores differ between groups known to be different on the variable of interest [18]. Hypothesis testing is the level to which measured values reflect the pre-defined hypotheses in terms of expected magnitude and direction of correlations or differences [19]. Reproducibility refers to the extent repeated measures (test-retest) provide similar results and is comprised of both reliability and agreement parameters [20]. Agreement concerns the intra individual difference between measurement (absolute measurement error) and reliability refers to the level of variance between two or more measurements that is due to the ‘true’ difference [19]. Responsiveness (longitudinal validity) is defined as the ability of a test to detect change over a time [20] and should be described in relation to the smallest detectable change. The smallest detectable change can be measured from test-retest studies provided the length of time between tests is appropriate and the variable being measured remains stable between tests [18]. It is important that the defined components of validity are investigated to understand the efficacy of tests and for researchers to make appropriate decisions on their use.

To determine the appropriateness of IMJTs to evaluate muscular strength and responsiveness to resistance exercise interventions, knowledge of validity properties is essential. Noting previous recommendations that validity is accumulated from multiple studies and cannot be demonstrated ‘once and for all’ by any single study [18], there is a clear requirement for a synthesis of evidence for IMJT. As such, the aims of this review were to determine the current evidence for IMJTs to assess muscular strength. We hypothesized that IMJT would have strong evidence in terms of reliability demonstrating intraclass correlation coefficient (ICC) ≥ 0.70. Additionally, criterion validity, construct validity and responsiveness to resistance exercise interventions would have moderate evidence supporting the validity of IMJT.

## Methods

The Preferred Reporting Items for Systematic Reviews and Meta-Analyses (PRISMA) guidelines were followed [21]. The updated Consensus-based Standards for the Selection of health Measurement Instruments (COSMIN) checklist [22] was applied as a method to critique the methodological quality and rating of measurement properties of individual research articles.

### Search Strategy

The PICO process (PICO acronym stands for P – patient, problem, population; I – intervention; C – comparison, control, comparator; O – outcome) was utilized to develop a search strategy based on the aims of the review. Initially the term ‘isometric multi-joint test’ was searched in Google Scholar, with the first 100 articles sorted by relevance and screened for variations in terminology within their titles, key words and text terms. All variations in terminology were recorded into a key terms list to enable the broadest possible search terms specifically relevant to the research question and aims of this review. A validated search method was adapted [23], and applied to search electronic databases; Web of Science, CINAHL and PubMed up to 18 March 2015 (Search terms and search example provided in Additional file 1). Searches with each electronic database search were amended for relevant index terms to reduce the potential of missing any relevant literature. Articles published in peer reviewed journals were considered based on title and abstract and checked for relevance. Secondly, full texts were retrieved and considered for inclusion against the established eligibility criteria. Articles that could not be retrieved electronically were accessed through the British Library Document Supply Service. Lastly, hand searching of article reference lists was completed to assess any articles not successfully retrieved by the search criteria. No time restrictions were applied to the literature search to ensure thoroughness of literature included. Following completion of literature searching, no further studies were considered for inclusion regardless of relevance.

### Eligibility Criteria

The following inclusion criteria were established based on the research question and agreed between authors:

I. ***Construct:*** studies with a lower body IMJT. Tests were maximal effort trials with the duration of the isometric test greater than 2 s to allow participants sufficient opportunity to achieve a true maximal force [9, 24].
II. ***Target population:*** studies involved healthy, uninjured, human participants between the ages of 15–65 years of age. Participants maintained normal nutritional habits throughout the duration of the study and were free from supplementation with any form of ergogenic aid.
III. ***Measurement instrument:*** testing was performed using verifiable isometric testing instruments, allowing direct measurement of vertical ground reaction force through a force plate. In accordance with the recommendations made by James et al. [25], force plate derived measurements should not be interchangeably compared to a load cell device to reduce potential for systematic error.
IV. ***Measurement properties:*** studies involved the evaluation of any measurement properties relating to the IMJT (e.g. reliability, validity, responsiveness, and interpretability).
V. ***Full text:*** Studies published in English. Adequate disclosure of information relating the status of participants and testing methods to enable assessment of methodological quality was required.

Relevant full-text articles were initially screened by DD to ensure inclusion criteria was met. The authors were familiar with the existing literature and had no conflicting bias with any of the literature screened for inclusion in the review. Agreement was required by two authors to exclude any study, a process previously outlined by Sampson et al. [26]. Two authors (DD and RK) independently rated included studies methodological quality and measurement property ratings. Authors resolved any rating disputes through open dialogue on how each study met the adapted COSMIN criteria until agreement was reached.

### Methodological Quality Evaluation

Included full-text articles underwent the following process of review. Firstly, for data extraction purposes, a study summary table was created outlining the number of participants and their strength training experience, description of performance test, measurement equipment and variables, level of familiarization and instruction, test position, as well as number of trials, duration and processing methods. Data extraction for studies included in the best evidence synthesis is presented in Table 1. All reviewed full-text articles are presented as author (year), title and journal in Additional file 2.

**Table 1:** Descriptive content of studies included in best evidence synthesis

Study	Training status participants (*n*) Age, years (mean or range) sex	Property of validity included	Performance test knee joint angle	No of trials duration of test (s) recovery between trials (minutes)	Processing of trials familiarization instruction
Alegre et al., (2006) [69]	Untrained *n* = 36 Age: 20.85 Male	Responsiveness	Isometric squat knee = 90°	Trials = not reported Duration = 4 Recovery = not reported	PRO = not reported FAM = not reported INS = hard and fast
Markovic and Jaric, (2004) [43]	Untrained *n* = 77 Age: Range 18–26 Male	Hypothesis	Isometric squat knee = 120°	Trials = 3 Duration = 5 Recovery = 2	PRO = best trial FAM = all participant’s familiar INS = gradually exert force until no further increase was detected
Markovic et al., (2007) [44]	Trained *n* = 93 Age: 20.1 Male	Reliability	Isometric squat knee = 120°	Trials = 3 Duration = not reported Recovery = not reported	PRO = best trial FAM = 1 week familiarization period INS = not reported
Markovic, (2007) [42]	Untrained *n* = 76 Age: 20.7 Male	Reliability	Isometric squat knee = 120°	Trials = 3 Duration = 5 Recovery = 2	PRO = not reported FAM = all participant’s familiar INS = not reported
Wilson et al., (1993) [74]	Untrained *n* = 41 Age: 22.5 Not reported	Responsiveness	Isometric squat knee = 135°	Trials = 2 Duration = 4 Recovery = 3	PRO = average of 2 trials FAM = 2 familiarization sessions INS = exert force in as short a time as possible
Crewther et al., (2012) [54]	Trained *n* = 79 Age: Range 18–32 Male	Hypothesis	IMTP knee = 135°	Trials = 2 Duration = 5 Recovery = 2	PRO = not reported FAM = not reported INS = hard and fast
Dos’Santos et al., (2015) [38]	Trained *n* = 30 Age: 17.5 Male	Reliability, Hypothesis	IMTP second pull position in the clean	Trials = 3 Duration = 5 Recovery = 1	PRO = best trial FAM = yes INS = hard and fast
Kraska et al., (2009) [40]	Untrained *n* = 63 Age: 19.9 Male and female	Reliability	IMTP knee 120–135°	Trials = 2 Duration = not reported Recovery = 1	PRO = average of best 2 FAM = not reported INS = hard and fast
Stone et al., (2004) [39]	Trained *n* = 50 Age: 31.0 Male and female	Reliability	IMTP knee 140–145°	Trials = 2–3 Duration = not reported Recovery = 2-3	PRO = average of best 2 FAM = not reported INS = hard and fast
West et al., (2011) [1]	Trained *n* = 39 Age: 24.0 Male	Reliability	IMTP knee 120–130°	Trials = not reported Duration = approx. 5 Recovery = not reported	PRO = not reported FAM = 1 session prior to testing INS = hard and fast

*IMTP* isometric mid-thigh pull, *PRO* processing of trials, *FAM* familiarization, *INS* instruction

Secondly, the methodological quality of each study was reviewed against the COSMIN four-point scoring criteria (excellent, good, fair, poor) whereby the worst score counts in each subsection. The authors made interpretive adaptations to the protocol and are outline accordingly below. Box A (internal consistency) and box E (structural validity) of the COSMIN procedure were deemed inappropriate for evaluation within this review as no statistical models were used to analyze data that was derived through the application of a questionnaire. Box F, Hypothesis were evaluated with specific relevance to multi-joint isometric tests only within each study. Box G, Cross-cultural validity was not evaluated, as none of the included studies were relevant to this section. Box H, criterion validity item 4, states can the criterion used is considered as a reasonable gold standard? This item was interpreted by defining the isoinertial repetition maximum (RM) squat as the current gold standard for evaluating resistance exercise. Box I, responsiveness item 7 states, was a proportion of the patients changed (i.e. improvement or deterioration)? This item was interpreted by the authors based on the magnitude of change in the group mean from baseline (*p* < .05) or effect size moderate or large relating only to isometric peak force measurements [27]. Measurement variables assessed within studies such as rates of force development or similar time-dependent variables were excluded from evaluation within this review. These variables represent a sub quality of strength as opposed to maximum muscular strength ability [28] and may be independently responsive to resistance exercise interventions [29].

Thirdly, measurement properties including reliability, measurement error, construct validity, criterion validity and responsiveness were evaluated in terms of the quantitative results for each included study using the criteria ‘positive’, ‘indeterminate’ and ‘negative’. In assessing measurement property rating for responsiveness, we defined smallest detectable change as the minimal change required to ensure the observed change is real [30]. Studies calculating standard error of measurement (SEM) were accepted as the smallest detectable change (SDC) and used for assessment. Interpretability was evaluated based on the requirement the study presented mean and standard deviation (SD) values for all groups relating to the isometric tests, allowing for extrapolation of study data.

The final stage of the review process was to synthesize the evidence, ‘a best evidence synthesis’ [31] performed by combining the evaluation of the methodological quality and the quality of measurement properties. Studies of fair or better methodological quality were included in the best evidence synthesis. The level of evidence was classified as “strong” when consistent findings of multiple good or at least one excellent study was present and the total sample size of eligible combined studies was ≥100, ‘moderate’ when consistent findings of multiple fair or at least one good study was present and the total sample size ≥50, “limited” when findings of at least one fair, good or excellent quality study was present and the total sample size between 25 and 49 and “unknown” when findings were of indeterminate rating, in studies with poor methodological quality or with a sample of ≤25 [32]. Where uncertainty in methodological approaches within studies was found during the review process, direct email correspondence was made based on the corresponding author details to request additional details concerning the study to ensure full evidence was examined.

### Classification of Training Status and Resistance Exercise Interventions

When reviewing the responsiveness of resistance exercise interventions, training status of participants is an important factor to consider given the magnitude of strength improvements differs considerably between untrained and trained individuals [33–35]. Classification of training status has been discussed in a review of strength training effects by Wernbom et al. [36]. These authors suggest a lack of studies exist involving participants of different training status and therefore these should be combined into one group, encompassing all ‘trained’, ‘advanced’ and ‘elite’ participants across studies. As such, this review operationally defined participant’s status into two groups, (i) untrained (less than 6 months resistance exercise experience) or (ii) trained, relating specifically to their resistance exercise experience being greater than 6 months.

Trainable characteristics of strength were classified as muscular strength, muscular hypertrophy and muscular power with respect to the primary sources of variation within resistance exercise programs. The variable components of resistance exercise programs determine the likely effect of interventions, these include exercise selection, exercise intensity, exercise volume, repetition speed/tempo and rest intervals. For a review, see Ratamess et al. [37].

## Results

**Fig. 1:**
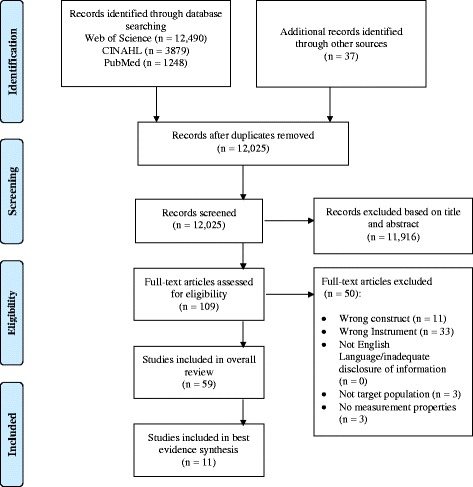
Flow chart of study selection and eligibility criteria

One-hundred nine full texts were reviewed with a total of fifty studies not meeting eligibility criteria for inclusion (see Fig. 1). A total of fifty-nine articles were analyzed within this review. Twenty-five studies investigated isometric squat, 31 investigated isometric mid-thigh pulls, 2 investigated isometric leg press and 1 investigated two IMJTs within the same study. The total number of participants across the included studies was 1394 with 59% having greater than 6 months’ resistance exercise experience. The mean ± SD age was 21.8 ± 2.7 with nine studies providing a range of ages to classify participants within their study and three studies not reporting age. Thirty-nine studies investigated male participants, 3 females only, 11 investigating a combination of male and female participants and six studies not disclosing the sex of participants. A total of 38 studies were included investigating trained participants with 23 in untrained participants. Two studies overlapped both trained and untrained participants (Table 2).

**Table 2:** Summary of best evidence synthesis

Type of test	Validity property	Level of evidence	Participants				Sex			
			Total	Trained	Untrained	Age, years (mean ± SD)	M	F	M and F	Unknown
Combined IMJT	Reliability	Strong	383	244	139	22.8 ± 4.71	4		3	
	Hypothesis	Strong	218	141	77	20.4 ± 3.19	3		1	
	Responsiveness	Moderate	78	0	78	21.9 ± 1.48	1			1
IMTP	Reliability	Moderate	182	119	63	23.1 ± 5.91	2		2	
	Hypothesis	Moderate	109	109	0	17.5 ± n/a	2			
	Responsiveness	Unknown								
Isometric squat	Reliability	Strong	201	125	76	22.3 ± 2.23	2		1	
	Hypothesis	Moderate	109	32	77	43.9 ± 2.65	1		1	
	Responsiveness	Moderate	78	0	78	21.9 ± 1.48	1			1

*IMJT* isometric multi-joint tests, *IMTP* isometric mid-thigh pull, *SD* standard deviation, *n/a* not applicable due to age range provided, *M* male, *F* female

### Measurement Properties

**Fig. 2:**
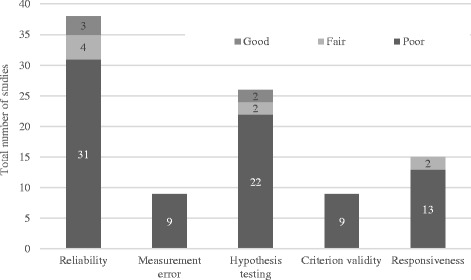
Rating distribution of included studies for methodological quality

Six of the nine measurement properties outlined in COSMIN were evaluated across the 59 studies that met the eligibility criteria. These properties were reliability (38 studies), measurement error (9 studies), hypothesis testing (26 studies), criterion validity (9 studies) and responsiveness (15 studies) (See Fig. 2). All included studies were deemed excellent in terms of content validity and therefore this property of validity is not discussed further within this review.

### Methodological Quality

#### Reproducibility

Reliability was assessed in thirty-eight included studies; three were rated as fair methodological quality for the isometric mid-thigh pull test [1, 38, 39] and one as good [40]. Reliability of the isometric squat test was rated fair in one study [41] and good in three studies [42–44]. Reliability of the isometric leg press was evaluated in one study [45] rated as poor methodological quality. The primary rationale for poor methodological quality in reliability studies was due to insufficient participant numbers. ICC reliability values for peak force in the seven noted studies ranged from ≥0.80 to 0.99 [1, 38–43], all rating positive in terms of measurement properties. Measurement error was evaluated in nine studies [15, 46–53] rated as poor methodological quality due to insufficient participant numbers to satisfy better rating classification.

#### Construct Validity and Hypothesis Testing

Twenty-six studies were rated; individual studies utilizing the isometric mid-thigh pull test were rated fair [38] and good [54]. Ratings for the isometric squat test of fair [41] and good [43] methodological quality were found within the included studies. All four studies performed hypothesis testing with one study additionally investigating construct validity for known positional groups in rugby union players [54]. Evaluation of measurement properties for construct validity revealed one study for the isometric squat test rated positive [41], one as indeterminate [43] with two other studies using the isometric mid-thigh pull test rating indeterminate [38, 54]. Twenty-two studies rated as poor in methodological quality [15, 16, 45–47, 50, 52, 53, 55–68] were limited due to insufficient participant numbers or lacked clarity in their hypotheses generation. Of these 22 studies, 7 rated positive [15, 45, 46, 50, 52, 56, 62] and 15 indeterminate [16, 47, 53, 55, 57–61, 63–68] for measurement properties.

#### Criterion Validity

Nine studies were rated as poor for methodological quality [14–16, 46, 50, 67, 69–71] due to insufficient participant numbers to satisfy a higher rating and one study did not assess the correlation between isometric squat and the criterion test 1RM squat [69]. Four studies of poor methodological quality [14, 16, 70, 71] investigated the isometric mid-thigh pull test and four investigated the isometric squat test [15, 46, 50, 69], with one further study investigating both the isometric mid-thigh pull and isometric squat test [67]. Seven studies of poor methodological quality were rated positively [14–16, 46, 50, 70, 71] in terms of methodological properties with correlation *≥*0.70 with the gold standard test, with one indeterminate [69] and one negative rating [67].

#### Responsiveness

In total, 15 studies were evaluated with only studies of poor methodological quality for isometric mid-thigh pull [24, 29, 60, 72, 73] found within included studies, two studies were rated fair for the isometric squat test [69, 74] and one as poor for the isometric leg press [45]. Both studies rated fair in methodological quality were rated as indeterminate for measurement properties due to no smallest detectable change being assessed. Studies assessed for intervention responsiveness varied in duration from 5 to 28 weeks, mean 10.6 ± 6.84 SD weeks and a mean frequency of 2.7 ± 0.916 SD sessions per week across interventions. Ten studies had longitudinal interventions [24, 29, 45, 46, 55, 60, 69, 72–74] and five studies investigated acute responses [41, 64, 75–77]. Included studies varied in resistance exercise protocol with Alegre et al. [69] using the half-squat exercise at an intensity between 50 and 60% 1RM for three sets of 6–10 reps. Significant changes in peak isometric force were found compared to the control group (*≥*4.77% increase, *d ≥* 0.11*, p ≤* 0*.*05). Wilson et al. [74] used the back-squat exercise, completing between 3 and 6 sets of 6–10 repetitions at a RM intensity in each respective set. Significant improvements were found in this study corresponding to increases in peak isometric force *≥*14.5% (*d ≥* 0.30, *p ≤* 0*.*05). Eight studies of poor methodological quality [24, 29, 45, 46, 55, 60, 72, 73] were limited by insufficient participant numbers to satisfy rating classification as fair in addition to four of the mentioned studies investigating acute responsiveness [41, 75–77], therefore not enabling assessment of longitudinal responsiveness.

#### Interpretability

Whilst not being rated within methodological quality rating, interpretability is an important characteristic of any measurement instrument across the included studies. Overall, 48 were rated positive and 11 as negative.

#### Best Evidence Synthesis

For the best evidence synthesis studies of fair or better methodological quality were considered and summarized for IMJTs combined. Additionally, the level of evidence for each individual isometric test is reported. No studies were included in the best evidence synthesis for either measurement error or criterion validity. No studies within the best evidence synthesis used the isometric leg press and therefore were classified as unknown in terms of the level of evidence supporting the use of this test.

With respect to reliability, there was strong evidence for combined IMJTs. There was moderate evidence for isometric mid-thigh pull and strong for isometric squat test. Evidence for hypothesis testing was found to be strong for combined IMJTs. Isometric mid-thigh pull and isometric squat tests individually have moderate level evidence supporting their use. Finally, there was moderate evidence for responsiveness of the isometric squat test and unknown evidence for the isometric mid-thigh pull and leg press test.

## Discussion

Assessment of physiological mechanisms and adaptations associated with resistance exercise is critical to improve understanding and efficacy of interventions [2]. The practical benefits of isometric multi-joint tests to assess resistance exercise interventions have been previously discussed [14, 15, 47, 56]. The aims of this review were to determine the level of evidence for IMJTs in their assessment of muscular strength.

Strong evidence was found for reliability of combined IMJTs including strong evidence for the isometric squat test and moderate evidence for the isometric mid-thigh pull independently. ICC values for reliability of peak force in the seven studies included in the best evidence synthesis ranging from ≥0.80 to 0.99, well above the acceptable threshold for ICC >0.7 as discussed in Baumgartner and Chung [78]. Researchers and practitioners can be confident with the reliability of IMJT measures of muscular strength. All best evidence synthesis inclusions within this review used a repeated trials design conducted on the same day. No studies to the authors knowledge used a day-to-day variability in measurements design (stability reliability) as defined by Baumgarter [79]. Atkinson and Nevill [80] have previously cautioned that exercise performance tests are affected by systematic bias. As such, reliability investigations may benefit from greater than one day between tests to get a true measurement on day to day variability. This review therefore highlights that stability reliability warrants further investigation, whereby studies using experimental designs account for day-to-day variability in measurements.

Our results demonstrate strong evidence to support construct validity (hypothesis testing) for combined isometric multi-joint tests, with moderate evidence for the isometric squat test and isometric mid-thigh pull, respectively, as independent tests. Primarily, the experimental design of three studies in the best evidence synthesis were correlational [38, 43, 54] with one study implementing an acute responsiveness design. No studies within the best evidence synthesis employed a study design assessing IMJTs discriminant validity, to investigate the difference between known groups. This is an additional aspect of validity that requires further exploration to fully understand the efficacy and application of IMJTs.

It was hypothesized that criterion validity would be supported with moderate level evidence based on knowledge of existing literature examining the relationship of isometric tests to dynamic performance tests. Additionally, Juneja et al. [81] suggests isometric strength testing has a strong potential to predict dynamic performance in strength based activities. Contrary to our hypothesis and previous suggestions [81], no appropriate evidence supporting the criterion validity of IMJTs was found. With nine studies in the overall review rated as poor methodological quality for criterion validity, none were accepted into the best evidence synthesis. Within this review, the isoinertial repetition maximum was defined as the gold standard comparison. As such, findings are not equally comparable with previous work by Juneja et al. [81] who evaluated criterion validity with various dynamic performance tests. This review highlights a paucity of evidence to support criterion validity of IMJTs relating to isoinertial repetition maximum performance. The current lack of evidence for criterion validity of IMJTs is due to eight studies within this review rated poor in methodological quality based on insufficient participant numbers to satisfy a higher rating. However, seven of these studies were rated positive for measurement properties with correlations *≥*0.70 with the gold standard test. This demonstrates a likelihood of strong evidence for criterion validity where future research investigating this critical component of validity satisfies key methodological criteria, such as appropriate participant numbers.

Moderate evidence was found supporting responsiveness for combined IMJTs in keeping with our hypothesis. Surprisingly this evidence was found only in studies using the isometric squat test [69, 74]. The principal reasons for studies not being included in the best evidence synthesis were due to insufficient participant numbers or absence of longitudinal interventions to assess responsiveness. Whilst multiple studies in this review [41, 63, 64, 76, 77] use acute response designs and receive poor methodological quality rating, they may have some generalizable merit for sports medicine and science readers. Given the resistance exercise intervention used by Alegre et al. [69] was classified as a muscular power intervention, only one study within the best evidence synthesis has examined responsiveness to a muscular strength intervention using high-intensity loading. Therefore, the use of moderate and high intensity loading schemes within resistance exercise interventions warrant further investigation to assess responsiveness of IMJTs.

Common methodological protocols for IMJTs are apparent amongst the best evidence inclusions (see Table. 1). Typically, studies use a 5-s test duration, 2–3 trials per testing session and between trials recovery time of 3 to 5 min. The instruction given to participants is consistently to push or pull as ‘hard and as fast as possible’ dependent on the type of the test, in all but one included study [43]. Methodological approaches to familiarization of participants, instruction around pre-tension and the processing of the trials was found to be variable within current literature. Joint angle at which the isometric test occurs is another methodological variation amongst studies, although inclusions within the best evidence synthesis in this review utilize a knee angle approximately 120° of flexion with one study using a 90° knee angle [69].

### Limitations

Whilst the COSMIN protocols have been applied in reviewing the methodological quality of performance tests in sports medicine and science research [32, 82, 83], they were not specifically designed for appraisal of performance tests. As such, several interpretive amendments were required by the authors as outlined in our methodology section. Participant numbers is a consistent limitation to study quality within the COSMIN protocols and may benefit further consideration given the tendency for sports medicine and science research to have relatively small participant groups. This is particularly the case in longitudinal studies where practical considerations in studying large participant numbers is an added challenge. Moreover, eligibility criteria were established to only include full-text articles published in English and therefore a possibility exists that appropriate research may have been missed due to publication in another language.

## Conclusions

IMJTs have been utilized as a measurement tool within 59 studies analyzed within this review. Researchers and sports practitioners based on strong evidence supporting their efficacy can confidently utilize isometric multi-joint tests with respect to reliability and construct validity. The findings of this review are generalizable to male, female, trained and untrained participants. IMJTs have demonstrated moderate responsiveness to resistance exercise. Future work to investigate this component of validity would further the understanding of current evidence. Despite the plethora of investigations examining critical aspects of validity, caution is urged in the application of IMJTs in relation to measurement error and criterion validity. Variability in test protocols must be carefully considered when interpreting IMJTs outcomes; therefore, authors are encouraged to provide comprehensive details on their respective testing protocols.

## Additional Files

Additional file 1: Search terms and search example. (DOCX 51 kb)

Additional file 2: Full text inclusion study table. (DOCX 59 kb)
