# Supplementary material for: The Validity and Responsiveness of Isometric Lower Body Multi-Joint Tests of Muscular Strength: a Systematic Review
Source: Sports Med Open. 2017 Jun 19;3:23. doi: 10.1186/s40798-017-0091-2 (PMC5476535; doi:10.1186/s40798-017-0091-2)
Supplement: Supplementary file 1 — Search terms and search example. (DOCX 51 kb) [file 40798_2017_91_MOESM1_ESM.docx]

**Title: The validity and responsiveness of isometric lower body multi–joint tests of muscular strength: A Systematic Review**

**Journal: Sports Medicine**

**Authors:** David Drake^1,2^, Rodney Kennedy^1^, Eric Wallace^3^

**Affiliations and addresses:**

^1^ School of Sport, Ulster University, Jordanstown, Shore Road, Newtownabbey, Co. Antrim, BT37 0QB, N. Ireland

^2^ Ulster Rugby, Kingspan Stadium, 134 Mount Merrion Avenue, Belfast, Co. Antrim, BT6 0FT, N. Ireland

**^3^** Sport and Exercise Sciences Research Institute, Ulster University, Jordanstown, Shore Road, Newtownabbey, Co. Antrim, BT37 0QB, N. Ireland

**Corresponding Author:**

David Drake

Email: [daviddrake87@gmail.com](mailto:daviddrake87@gmail.com)

Electronic Supplementary Material Appendix S1: Search terms and search example

***Identified key terms***

1. Muscular strength
2. Peak force
3. Max* strength
4. Force*time
5. Resistance training
6. Weightlifting
7. Adaptation
8. Physiological
9. multi*joint
10. leg press
11. squat
12. mid thigh pull
13. multi*movement
14. Isometric

***Web of science search***

Key terms 1 OR 2 OR 3 OR 4 OR 5 OR 6 OR 7 OR 8 OR 9 OR 10 OR 11 OR 12 OR 13 AND 14

Results refined by language (English Only) and document types article or report or clinical trial or unspecified.

*Timespan=All years*

*Search language=Auto*

12490 results returned
