# Supplementary material for: The Validity and Responsiveness of Isometric Lower Body Multi-Joint Tests of Muscular Strength: a Systematic Review
Source: Sports Med Open. 2017 Jun 19;3:23. doi: 10.1186/s40798-017-0091-2 (PMC5476535; doi:10.1186/s40798-017-0091-2)
Supplement: Supplementary file 2 — Full text inclusion study table. (DOCX 59 kb) [file 40798_2017_91_MOESM2_ESM.docx]

**Title: The validity and responsiveness of isometric lower body multi–joint tests of muscular strength: A Systematic Review**

**Journal: Sports Medicine**

**Authors:** David Drake^1,2^, Rodney Kennedy^1^, Eric Wallace^3^

**Affiliations and addresses:**

^1^ School of Sport, Ulster University, Jordanstown, Shore Road, Newtownabbey, Co. Antrim, BT37 0QB, N. Ireland

^2^ Ulster Rugby, Kingspan Stadium, 134 Mount Merrion Avenue, Belfast, Co. Antrim, BT6 0FT, N. Ireland

**^3^** Sport and Exercise Sciences Research Institute, Ulster University, Jordanstown, Shore Road, Newtownabbey, Co. Antrim, BT37 0QB, N. Ireland

**Corresponding Author:**

David Drake

Email: [daviddrake87@gmail.com](mailto:daviddrake87@gmail.com)

Electronic Supplementary Material Appendix S2: Full text inclusion study table

| **Author (Year) [In text reference number]** | **Article Title** | **Journal** |
| --- | --- | --- |
| Alegre, L. M., Jiménez, F., Gonzalo-Orden, J. M., Martín-Acero, R. and Aguado, X. (2006) [69] | Effects of dynamic resistance training on fascicle length and isometric strength | Journal of Sports Sciences |
| Bazyler, C.D., Bailey, C.A., Chiang, C., Sato, K., Stone, M. (2014) [55] | The effects of strength training on isometric force production symmetry in recreationally trained males. | Journal of Trainology |
| Bazyler, C. D., Sato, K., Wassinger, C. A., Lamont, H. S., Stone, M. H. (2014) [46] | The efficacy of incorporating partial squats in maximal strength training | J Strength Cond Res |
| Bazyler, C. D., Beckham, G. K. and Sato, K. (2015) [15] | The use of the isometric squat as a measure of strength and explosiveness | J Strength Cond Res |
| Beckham, G., Lamont, H.S., Sato, K., Ramsey, M.W., Haff, G., Stone, M. (2012) [no in text reference] | Isometric strength of powerlifters in key positions of the conventional deadlift. | Journal of Trainology |
| Beckham, G., Mizuguchi, S., Carter, C., Sato, K., Ramsey, M., Lamont, H., Hornsby, G., Haff, G. and Stone, M. (2013) [56] | Relationships of isometric mid-thigh pull variables to weightlifting performance | J Sports Med Phys Fitness |
| Blazevich, A. J., Gill, N. and Newton, R. U. (2002) [50] | Reliability and validity of two isometric squat tests | J Strength Cond Res |
| Comfort, Paul Graham-Smith, Phillip Matthews, Martyn J. Bamber, Chris. (2011) [no in text reference] | Strength and power characteristics in English elite rugby league players | J Strength Cond Res |
| Comfort, P., Jones, P. A., McMahon, J. J. and Newton, R. (2015) [47] | Effect of knee and trunk angle on kinetic variables during the isometric mid-thigh pull: test-retest reliability | Int J Sports Physiol Perform |
| Cormie, P., Deane, R. S., Triplett, N. T. and McBride, J. M. (2006) [57] | Acute effects of whole-body vibration on muscle activity, strength, and power | J Strength Cond Res |
| Crewther, B. T., Kilduff, L. P., Cook, C. J., Cunningham, D. J., Bunce, P. J., Bracken, R. M. and Gaviglio, C. M. (2012) [54] | Scaling strength and power for body mass differences in rugby union players | J Sports Med Phys Fitness |
| Dos'Santos, T., Jones, P. A., Kelly, J., McMahon, J. J., Comfort, P. and Thomas, C. (2016) [38] | Effect of Sampling Frequency on Isometric Mid-Thigh Pull Kinetics | Int J Sports Physiol Perform |
| Dumke, C. L., Pfaffenroth, C. M., McBride, J. M. and McCauley, G. O. (2010) [58] | Relationship between muscle strength, power and stiffness and running economy in trained male runners | Int J Sports Physiol Perform |
| Granacher, U., Muehlbauer, T., Doerflinger, B., Strohmeier, R. and Gollhofer, A. (2011) [45] | Promoting strength and balance in adolescents during physical education: effects of a short-term resistance training | J Strength Cond Res |
| Haff, G. Gregory, Stone, Michael, O'Bryant, Harold S., Harman, Everett, Dinan, Chris, Johnson, Robert and Han, Ki-Hoon. (1997) [61] | Force-Time Dependent Characteristics of Dynamic and Isometric Muscle Actions | J Strength Cond Res |
| Haff, G. G., Carlock, J. M., Hartman, M. J., Kilgore, J. L., Kawamori, N., Jackson, J. R., Morris, R. T., Sands, W. A. and Stone, M. H. (2005) [59] | Force-time curve characteristics of dynamic and isometric muscle actions of elite women olympic weightlifters | J Strength Cond Res |
| Haff, G. G., Jackson, J. R., Kawamori, N., Carlock, J. M., Hartman, M. J., Kilgore, J. L., Morris, R. T., Ramsey, M. W., Sands, W. A. and Stone, M. H. (2008) [60] | Force-time curve characteristics and hormonal alterations during an eleven-week training period in elite women weightlifters | J Strength Cond Res |
| Haff, G. G., Ruben, R. P., Lider, J., Twine, C. and Cormie, P. (2015) [no in text reference] | A comparison of methods for determining the rate of force development during isometric mid-thigh clean pulls | J Strength Cond Res |
| Izquierdo, M., Aguado, X., Ribas, T., Linares, F., Vila, L., Voces, J. A., Alvarez, A. I. and Prieto, J. G. (1998) | Jumping performance, isometric force and muscle characteristics in non-athletic young men | Journal of Human Movement Studies |
| Kawamori, N., Rossi, S. J., Justice, B. D., Haff, E. E., Pistilli, E. E., O'Bryant, H. S., Stone, M. H. and Haff, G. G. (2006) [62] | Peak force and rate of force development during isometric and dynamic mid-thigh clean pulls performed at various intensities | J Strength Cond Res |
| Khamoui, A. V., Brown, L. E., Nguyen, D., Uribe, B. P., Coburn, J. W., Noffal, G. J. and Tran, T. (2011) | Relationship between force-time and velocity-time characteristics of dynamic and isometric muscle actions | J Strength Cond Res |
| Kraska, J. M., Ramsey, M. W., Haff, G. G., Fethke, N., Sands, W. A., Stone, M. E. and Stone, M. H. (2009) [40] | Relationship between strength characteristics and unweighted and weighted vertical jump height | Int J Sports Physiol Perform |
| Lawton, Trent W., Cronin, John B., McGuigan, Mike R. (2012) [72] | Does extensive on-water rowing increase muscular strength and endurance? | Journal of Sports Sciences |
| Leary, B. K., Statler, J., Hopkins, B., Fitzwater, R., Kesling, T., Lyon, J., Phillips, B., Bryner, R. W., Cormie, P. and Haff, G. G. (2012) [63] | The relationship between isometric force-time curve characteristics and club head speed in recreational golfers | J Strength Cond Res |
| Linnamo, V., Newton, R. U., Hakkinen, K., Komi, P. V., Davie, A., McGuigan, M. and Triplett-McBride, T. (2000) [75] | Neuromuscular responses to explosive and heavy resistance loading | Journal of Electromyography and Kinesiology |
| Markovic, G. and Jaric, S. (2004) [43] | Movement performance and body size: the relationship for different groups of tests | Eur J Appl Physiol |
| Markovic, G. Jukic, I. Milanovic, D. Metikos, D. (2007) [44] | Effects of sprint and plyometric training on muscle function and athletic performance | J Strength Cond Res |
| Markovic, G. (2007) [42] | Poor relationship between strength and power qualities and agility performance | J Sports Med Phys Fitness |
| Marshall, P. W., Robbins, D. A., Wrightson, A. W., Siegler, J. C. (2012) [64] | Acute neuromuscular and fatigue responses to the rest-pause method | J Sci Med Sport |
| McBride, J. M., Cormie, P. and Deane, R. (2006) [65] | Isometric squat force output and muscle activity in stable and unstable conditions | J Strength Cond Res |
| McBride, J. M., Deane, R. and Nimphius, S. (2007) [66] | Effect of stretching on agonist-antagonist muscle activity and muscle force output during single and multiple joint isometric contractions | Scand J Med Sci Sports |
| McCaulley, G. O., McBride, J. M., Cormie, P., Hudson, M. B., Nuzzo, J. L., Quindry, J. C. and Travis Triplett, N. (2009) [76] | Acute hormonal and neuromuscular responses to hypertrophy, strength and power type resistance exercise | Eur J Appl Physiol |
| McGuigan, M. R., Winchester, J. B. and Erickson, T. (2006) [71] | The importance of isometric maximum strength in college wrestlers | Journal of Sports Science and Medicine |
| McGuigan, M. R. and Winchester, J. B. (2008) [14] | The relationship between isometric and dynamic strength in college football players | Journal of Sports Science and Medicine |
| McGuigan, M. R., Newton, M. J., Winchester, J. B. and Nelson, A. G. (2010) [70] | Relationship between isometric and dynamic strength in recreationally trained men | J Strength Cond Res |
| Nicholson, G., McLoughlin, G., Bissas, A. and Ispoglou, T. (2014). [77] | Do the acute biochemical and neuromuscular responses justify the classification of strength- and hypertrophy-type resistance exercise? | J Strength Cond Res |
| Nuzzo, J. L., McBride, J. M., Cormie, P. and McCaulley, G. O. (2008) [67] | Relationship between countermovement jump performance and multijoint isometric and dynamic tests of strength | J Strength Cond Res |
| Rahmani, A., Viale, F., Dalleau, G. and Lacour, J. R. (2001) [48] | Force/velocity and power/velocity relationships in squat exercise | Eur J Appl Physiol |
| Sheppard, J. M., Nimphius, S., Haff, G. G., Tran, T. T., Spiteri, T., Brooks, H., Slater, G. and Newton, R. U. (2013) [49] | Development of a comprehensive performance-testing protocol for competitive surfers | Int J Sports Physiol Perform |
| Spiteri, T., Nimphius, S., Hart, N. H., Specos, C., Sheppard, J. M. and Newton, R. U. (2014) [16] | Contribution of strength characteristics to change of direction and agility performance in female basketball athletes | J Strength Cond Res |
| Stone, M. H., Sanborn, K., O'Bryant, H. S., Hartman, M., Stone, M. E., Proulx, C., Ward, B. and Hruby, J. (2003) [73] | Maximum strength-power-performance relationships in collegiate throwers | J Strength Cond Res |
| Stone, M. H., Sands, W. A., Carlock, J., Callan, S., Dickie, D., Daigle, K., Cotton, J., Smith, S. L. and Hartman, M. (2004) [39] | The importance of isometric maximum strength and peak rate-of-force development in sprint cycling | J Strength Cond Res |
| Stone, M. H., Sands, W. A., Pierce, K. C., Carlock, J., Cardinale, M. and Newton, R. U. (2005) [no in text reference] | Relationship of maximum strength to weightlifting performance | Med Sci Sports Exercise |
| Stone, M. H., Sands, W. A., Pierce, K. C., Ramsey, M. W. and Haff, G. G. (2008) [no in text reference] | Power and power potentiation among strength-power athletes: preliminary study | Int J Sports Physiol Perform |
| Storey, A., Wong, S., Smith, H. K. and Marshall, P. (2012) [41] | Divergent muscle functional and architectural responses to two successive high intensity resistance exercise sessions in competitive weightlifters and resistance trained adults | Eur J Appl Physiol |
| Teo, W. P., McGuigan, R. and Newton, M. J. (2011) [68] | The effects of circadian rhythmicity of salivary cortisol and testosterone on maximal isometric force, maximal dynamic force, and power output | J Strength Cond Res |
| Thomas, C., Comfort, P., Chiang, C., Jones, P.A. (2015) [51] | Relationship between isometric mid thigh pull variables and sprint and change of direction performance in collegiate athletes | Journal of Trainology |
| Thomas, C., Jones, P. A. and Comfort, P. (2015) [52] | Reliability of the Dynamic Strength Index in Collegiate Athletes | Int J Sports Physiol Perform |
| Thomas, C., Jones, P. A., Rothwell, J., Chiang, C. Y. and Comfort, P. (2015) [53] | An Investigation into the Relationship Between Maximum Isometric Strength and Vertical Jump Performance | J Strength Cond Res |
| Tillin, N. A., Pain, M. T. G. and Folland, J. (2013) [3] | Explosive force production during isometric squats correlates with athletic performance in rugby union players | Journal of Sports Sciences |
| Utter, Alan, Stone, Michael, O'Bryant, Harold, Summinski, Richard and Ward, Barrymore. (1998) [24] | Sport-Seasonal Changes in Body Composition, Strength, and Power of College Wrestlers | J Strength Cond Res |
| Verdera, F., Champavier, L., Schmidt, C., Bermon, S. and Marconnet, P. (1999) [7] | Reliability and validity of a new device to measure isometric strength in polyarticular exercises | J Sports Med Phys Fitness |
| West, D. J., Owen, N. J., Jones, M. R., Bracken, R. M., Cook, C. J., Cunningham, D. J., Shearer, D. A., Finn, C. V., Newton, R. U., Crewther, B. T. and Kilduff, L. P. (2011) [1] | Relationships between force-time characteristics of the isometric mid-thigh pull and dynamic performance in professional rugby league players | J Strength Cond Res |
| Whittington, J., Schoen, E., Labounty, L. L., Hamdy, R., Ramsey, M. W., Stone, M. E., Sands, W. A., Haff, G. G. and Stone, M. H. (2009) [no in text reference] | Bone mineral density and content of collegiate throwers: influence of maximum strength | J Sports Med Phys Fitness |
| Wilson, G. J., Newton, R. U., Murphy, A. J. and Humphries, B. J. (1993) [74] | The optimal training load for the development of dynamic athletic performance | Medicine and Science in Sports and Exercise |
| Wilson, G. J. and Murphy, A. J. (1996) [no in text reference] | Strength diagnosis: The use of test data to determine specific strength training | Journal of Sports Sciences |
| Winchester, J. B., McBride, J. M., Maher, M. A., Mikat, R. P., Allen, B. K., Kline, D. E. and McGuigan, M. R. (2008) [29] | Eight weeks of ballistic exercise improves power independently of changes in strength and muscle fiber type expression | J Strength Cond Res |
| Young, W., McLean, B. and Ardagna, J. (1995) [17] | Relationship between strength qualities and sprinting performance | J Sports Med Phys Fitness |
| Young, W., Wilson, G. and Byrne, C. (1999) [no in text reference] | Relationship between strength qualities and performance in standing and run-up vertical jumps | J Sports Med Phys Fitness |
